# Screening of at‐risk blood donors for Chagas disease in non‐endemic countries: Lessons from a 2‐year experience in Tuscany, Italy

**DOI:** 10.1111/tme.12741

**Published:** 2020-12-08

**Authors:** Valentina D. Mangano, Marco Prato, Antonella Marvelli, Giovanna Moscato, Fabrizio Bruschi

**Affiliations:** ^1^ Department of Translational Research N.T.M.S., Università di Pisa Pisa Italy; ^2^ Department of Laboratory Medicine Unit of Microbiology, Azienda Ospedaliera Universitaria Pisana Pisa Italy; ^3^ Postgraduate School of Microbiology and Virology Università di Pisa Pisa Italy; ^4^ Postgraduate School of Clinical Pathology and Biochemistry Università di Pisa Pisa Italy; ^5^ Department of Laboratory Medicine Unit of Clinical Chemistry Analyses, Azienda Ospedaliera Universitaria Pisana Pisa Italy; ^6^ Department of Laboratory Medicine Programme of Parasitic Disease monitoring, Azienda Ospedaliera Universitaria Pisana Pisa Italy; ^7^ Present address: Department of Surgical, Medical and Molecular Pathology and Critical Care Medicine Università di Pisa Pisa Italy

**Keywords:** blood donors, Chagas disease, screening, serology, transfusion

## Abstract

**Background:**

Chagas disease (CD) is caused by the protozoan parasite *Trypanosoma cruzi* and is transmitted by blood‐sucking triatomine insects in endemic areas of Latin America. Transmission can also occur via blood transfusion and is a major cause of CD in non‐endemic areas.

**Objectives:**

The aim of the study was to assess the prevalence of anti‐*T. cruzi* antibodies in blood donors at risk of infection in Tuscany, Italy, following the introduction of blood safety Italian legislation.

**Material and methods:**

Donors (*N* = 1985) were tested in 2016 to 2018 for anti‐*T. cruzi* IgG using an immunochromatographic test (ICT). Chemiluminescent immunoassay (CLIA) was performed on ICT‐positive donors to exclude CD, whereas enzyme‐linked immunosorbent assay and western blot were performed in case of discordant results. All assays were performed on CD patients (*N* = 10) for validation.

**Results:**

Ten blood donors had a positive ICT result, with a resulting *T. cruzi* seroprevalence of 0.5% but demonstrated negative results to CLIA, as well as to the other serological assays. The comparison of serological assays suggested a lower relative sensitivity of ICT.

**Conclusion:**

The results of this study confirm the significance of serological testing in the screening strategy for CD. However, they provide evidence for discontinuing the use of ICT as a screening test and suggest that a sensitive, specific and multi‐sample format assay should be used at the national level for uniformity of results.

## 1 INTRODUCTION

Chagas disease (CD), or American trypanosomiasis, is caused by the protozoan parasite *Trypanosoma cruzi* and is transmitted by blood‐sucking triatomine insects (mainly *Triatoma*, *Panstrongylus*, *Rhodnius*) in endemic areas of continental Latin America.

Transmission of the parasite can also occur vertically from mother to child, via transfusion with blood and blood products, through the transplant of solid organs or haematopoietic stem cells, consumption of food or drinks contaminated by vectors or their faeces and via laboratory accidents.^1^

The acute phase of the infection, although mostly asymptomatic, may present with fever, inflammation at the inoculation site (chagoma), unilateral palpebral oedema (Romaña sign), lymphadenopathy and hepatosplenomegaly. After 4–6 weeks, the acute phase resolves spontaneously, and blood parasitaemia reduces substantially, but patients remain chronically infected, and in 30%–40% of them, chronic disease may occur up to 30 years later, with cardiomyopathy and/or megaviscera as the most common clinical manifestations.

In endemic countries, due to multinational control initiatives, the epidemiological situation has dramatically improved, with a significant decline in the incidence of the disease in the last decade. For example, in Argentina, one of the most affected countries in Latin America, a decrease of the infected population has been estimated from 23.0% in 2005 to 5.2% in 2010.^1^

In contrast, in non‐endemic countries, a dramatic increase in the number of CD cases has been reported in the last decades, changing the scenario of the disease into a worldwide public health concern.^2^

For example, in the United States and in Canada, more than 325 000 and between 4000 and 6000 cases of CD have been estimated, respectively. As for Europe, in Spain and Italy, the two countries with the highest number of Latin American immigrants, around 70 000 to 76 000 and 90 000 to 10 000 infected individuals have been estimated, respectively.^1^

According to the World Health Organization (WHO), for the diagnosis of CD, at least two serological tests based on different principles should be performed to detect anti‐*T. cruzi* antibodies.^3^

Different strategies have been adopted in order to reduce the risk of transfusion‐transmitted (TT) CD. Currently, in endemic areas, all donations should be analysed for *T. cruzi* antibodies. In non‐endemic countries, interventions are different: exclusion of at‐risk donors (Sweden), *T. cruzi* serology screening of at‐risk blood donors (Portugal, Spain, France and—more recently—Italy) or one‐time serological testing of all donors (United States)^4^ (Angheben, personal communication). Where serological screening is performed, a single test is considered sufficient to decide on exclusion from donation.^1^ For its part, the WHO recommends the use of a single enzyme‐linked immunosorbent assay (ELISA) for blood bank screening.^3^

The aim of this study was to assess the seroprevalence of *T. cruzi*‐specific antibodies in a population of at‐risk blood donors from Tuscany, Italy, in a 2‐year period following the introduction of the Italian National Blood Centre regulation in November 2015 (L.219, D.M. 02/11/2015). This requires the identification of at‐risk candidate donors (those born in Latin America, born from Latin American mother or travellers with history of rural or outdoor activities in endemic areas) through a questionnaire, followed by testing for anti‐T. cruzi antibodies with immunological techniques, without any further specification on the characteristics of the test itself. Therefore, samples from both at‐risk blood donors and CD patients were tested with different serological methods in order to provide evidence for the amelioration of the current screening strategy.

## 2 METHODS

### 2.1 *Study area and population*

#### 2.1.1 Blood donors at‐risk for *T. cruzi* infection

The Parasitology laboratory at Pisa University Hospital is responsible for the serological testing of serum samples collected from blood donors identified as at risk for *T. cruzi* infection by the Transfusion Centres of the Provinces of Livorno, Lucca, Massa‐Carrara and Pisa (northwest area of the Tuscany Regional health system). At‐risk blood donors were identified through a questionnaire and serologically tested, as required by the current legislation. Between 15 February 2016 and 31 December 2018 the laboratory has performed *n* = 2042 immunochromatographic (ICT) tests (Chagas Quick Test, Cypress Diagnostics, Belgium; the manufacturer reports 100% sensitivity and 100% specificity based on 91 sera samples from Chile and comparison with ELISA and IFA assays) on serum samples from *n* = 1985 candidate blood donors. The choice of serological method used was constrained by availability for acquisition through the Tuscany regional health system (ESTAR). ICT was therefore used despite the WHO not recommending the use of rapid tests for blood‐screening purposes.^3^

#### 2.1.2 Samples from CD patients

The Department of Infectious—Tropical Medicine and Microbiology of the IRCCS Sacro Cuore Don Calabria Hospital in Negrar, Verona, has an in‐ and out‐patient service, and more than 400 individuals have been diagnosed and offered treatment for CD in the last decades. Samples of blood from 10 patients born in Bolivia who were recruited during screening surveys of Latin American communities between 2012 and 2015 and underwent serology diagnosis for CD through two ELISA assays (details provided below) have been selected as controls for the current study. Nine individuals tested positive to both tests, chronic CD was diagnosed (indeterminate phase), and they were therefore used as positive controls. One patient tested negative for both tests and was therefore considered a negative control.

### 2.2 *Serological methods*

Six different assays currently present in the Italian market were performed for the detection of anti‐*T. cruzi* antibodies in serum samples from ICT‐seropositive candidate donors, as well as from control patients, to compare the performance of methods.

#### 2.2.1 Immunochromatographic test

The ICT method Chagas Quick Test (Cypress Diagnostics) was performed according to manufacturer's instructions. The ICT has a strip format, and it is based on *T. cruzi* recombinant antigen including nine different epitopes and requires 10 μl of serum. After 30 min, results can be read on the strip. A sample is considered positive if both test and control lines appear on the strip, negative if only the control line is present and invalid if the control line is absent.

#### 2.2.2 Chemiluminescent immunoassay

The chemiluminescent immunoassay (CLIA) method Architect System Chagas (Abbott) was performed following manufacturer's instructions using the instrument Architect i2000 SR (Abbott). The CLIA is based on four recombinant proteins of *T. cruzi* (FP3, FP6, FP10, TcF) and requires 100 μl of serum. At the end of the reaction (∼60 min), the chemiluminescent signal (relative light units, RLUs) is read. A calibrator sample is assayed along with the test samples, and a cut‐off value (CO) is obtained as the mean of three values of the calibrator. The positivity is defined by the ratio between the RLUs of the sample (S) and the RLUs of the cut‐off (CO). A sample is considered positive if S/CO ≥ 1, negative if S/CO ≤ 0.8 and uncertain if 0.8 < S/CO < 1.

#### 2.2.3 Enzyme‐linked immunosorbent assay 1

The ELISA method Chagatest ELISA lisado (Wienerlab) was performed according to manufacturer's instructions. The ELISA has a 96‐well plate format; it is based on *T. cruzi* lysate antigen and requires 20 μl of serum. At the end of the reaction (∼180 min), the absorbance (OD) is measured using a spectrophotometer (Seac ELISA‐Reader Sirio S, Radim Diagnostic) at a wavelength of 450 nm. A negative control sample is assayed along with test samples, and a CO is obtained as the mean of three negative control values +0.200. A sample is considered positive if OD ≥ CO and negative if OD < CO.

#### 2.2.4 Enzyme‐linked immunosorbent assay 2

The ELISA method Bioelisa Chagas (Biokit) was performed following manufacturer's instructions. The ELISA has a 96‐well plate format, uses a *T. cruzi* recombinant antigen including four immunodominant epitopes and requires 10 μl of serum. At the end of the reaction (∼180 min), the absorbance (OD) is measured using a spectrophotometer (Seac ELISA‐Reader Sirio S, Radim Diagnostic) at a wavelength of 450 nm. A negative control sample is assayed along with test samples, and CO is obtained as the mean of three negative control values +0.300. A ratio between the samples' OD (S) and the CO is calculated. A sample is considered positive if the S/CO ratio is equal or greater than 1, negative if the ratio is lower than 0.9 and uncertain if the ratio is in the 0.9–1.0 range.

#### 2.2.5 Western blot 1

The Western blot (WB) method Chagas IgG Lineblot (Novatec) was performed according to manufacturer's instructions. The WB, in a strip format is based on *T. cruzi* TcF recombinant antigen and requires 10 μl of serum. At the end of the reaction (∼180 min), results were read with the naked eye on the strip. The strip contains three control lines: Sample Load Control (SLC), Conjugate Control (CC) and CO; the assay is valid if the colour intensity of the control lines is SLC ≥ CC > CO. A sample is considered positive if the intensity of the test line is greater than CO, negative if the intensity of the test line is lower than CO and uncertain if the intensity of the test line is equal to CO.

#### 2.2.6 Western blot 2

The WB method Chagas Western Blot IgG (LDBIO Diagnostic) was performed according to manufacturer's instructions. The WB, in a strip format, is based on *T. cruzi* lysate antigens and requires 10 μl of serum. A positive control sample is assayed along with test samples to assess the validity of the assay. At the end of the reaction (∼3.5 h), results were read with the naked eye on the strip, according to manufacturer's instructions. A sample is considered positive if at least two clearly defined bands among five couples of bands (P15‐16, P21‐22, P27‐28, P42, P45‐47) in the molecular weight range of 10–200 kDa were present. A sample is considered negative if the two bands were absent.

## 3 RESULTS

In the 2016–2018 period, 1985 blood donors at risk for *T. cruzi* infection were identified through a questionnaire at transfusion centres in the northwest area of Tuscany. The average proportion of at‐risk blood donors in the whole population was 2.4% in this region (personal communication from the directors of the transfusion centres). The number of blood donors at risk whose sera samples were tested for anti‐*T. cruzi* antibodies were: 985 in 2016, 512 in 2017 and 488 in 2018, with 216 from Livorno, 340 from Lucca, 564 from Massa‐Carrara, 233 from Pisa and 597 from Viareggio.

Ten blood donors had a positive result at the screening test (ICT), with a resulting *T. cruzi* seroprevalence of 0.5% (*N* = 10/1985, 95% CI = 0.3%–0.9%). The seropositive subjects were excluded from blood donation, in agreement with the current legislation. The introduction of laboratory testing in the screening strategy has therefore resulted in safe blood donation from 1975 healthy subjects, with an impressive reduction in the potential loss of blood supply.

Table 1 shows the distribution of demographic characteristics (gender, age group, country of birth) among the total population of at‐risk donors screened and among the group of donors with a seropositive result, as well as the frequency of seropositive donors. Females, individuals aged 18–29 years old and individuals born in Latin American countries seem over‐represented among seropositive donors with respect to the total population of donors at risk, although differences in seropositivity are not statistically significant.

**TABLE 1:** Characteristics of blood donors at risk for *Trypanosoma cruzi* infection and of *T. cruzi* seropositive blood donors

Demographic characteristic		*N* donors at risk (%)	*N* seropositive donors (%)	% Seropositivity (95% CI)
Gender	Female	786 (39.6)	6 (60.0)	0.8 (0.4–1.7)
	Male	1199 (60.4)	4 (40.0)	0.3 (0.1–0.9)
Age group (years)	18–29	361 (18.2)	5 (50.0)	1.4 (0.6–3.2)
	30–39	435 (21.9)	1 (10.0)	0.2 (0.0–1.3)
	40–49	563 (28.4)	3 (30.0)	0.5 (0.2–1.6)
	50–69	439 (22.1)	0 (0.0)	0.0 (0.0–0.9)
	60–71	156 (7.9)	1 (10.0)	0.6 (0.1–3.5)
	Unknown	31 (1.6)	0 (0.0)	0.0 (0.0–11.0)
Continent of birth	Europe	1534 (77.3)	8 (80.0)	0.5 (0.3–1.0)
	Latin America	205 (10.3)	2 (20.0)	1.0 (0.3–3.5)
	Other	19 (1.0)	0 (0.0)	0.0 (0.0–16.8)
	Unknown	227 (11.4)	0 (0.0)	0.0 (0.0–1.7)
Total		1985 (100)	10 (100)	0.5 (0.3–0.9)

*Note:* The table shows the number (*N*) and percentage (%) of study subjects stratified by gender, age group and country of birth in the total population of blood donors at risk of *T. cruzi* infection and in the group of donors seropositive for anti‐*T. cruzi* antibodies, together with the percentage of seropositive individuals (% Seropositivity) and its 95% CI.

A second test (CLIA) is performed on sera samples of ICT‐seropositive subjects as part of the diagnostic algorithm for chronic CD. All 10 subjects with an ICT‐positive result had a CLIA‐negative result.

WHO guidelines for the diagnosis of chronic CD recommend performing a third serological test in case of discordance of the first and second tests.^3^ As it is not yet established which assay should be used as a confirmatory test, all commercially available assays at the time of the study were performed for evaluation: two different ELISA tests and two different WB tests (Table 2).

**TABLE 2:** Results of different serological methods for the detection of *Trypanosoma cruzi* antibodies in ICT‐seropositive blood donors

Donor ID	ICT result	CLIA result	CLIA index	ELISA1 result	ELISA1 index	ELISA2 result	ELISA2 index	WB1 result	WB2 result
2716	pos	neg	0.02	neg	0.11	neg	0.02	neg	neg
2190	pos	neg	0.01	neg	0.67	neg	0.04	pos	neg
4190	pos	neg	0.01	…	…	…	…	neg	neg
3189	pos	neg	0.15	neg	0.20	neg	0.05	neg	neg
9472	pos	neg	0.09	neg	0.84	neg	0.05	neg	neg
1180	pos	neg	0.02	…	…	…	…	neg	neg
7190	pos	neg	0.07	neg	0.19	neg	0.06	neg	neg
3971	pos	neg	0.01	neg	0.21	neg	0.01	neg	neg
1597	pos	neg	0.02	neg	0.06	neg	0.20	neg	neg
2993	pos	neg	0.06	neg	0.08	neg	0.08	neg	neg

*Note:* The table shows the results of different serological methods for the detection of *T. cruzi* antibodies in ICT‐positive, at‐risk blood donors (*N* = 10). “…” denotes data not available because of lack of sera.

Abbreviations: ELISA1, ELISA using recombinant antigens; ELISA2, ELISA using lysate antigen; WB1, WB using TcF recombinant antigen; WB2, WB using lysate antigen.

Both ELISA tests and the WB test based on *T. cruzi* lysate antigen gave a negative result for all sera samples, whereas the WB test based on TcF recombinant antigen gave a negative result for nine samples and a positive result for one sample. Taken together, these results indicate that, there is no evidence of chronic CD for any of the at‐risk blood donors with a positive ICT result for *T. cruzi* antibodies.

To evaluate the concordance and relative sensitivity of the different serological tests (ICT, CLIA, ELISA1, ELISA2, WB1, WB2), those were performed on control sera samples from CD patients (*N* = 10). One patient had a negative result for all tests, and eight patients had a positive result for all tests, whereas one patient had a positive result for all tests but ICT (Table 3). These results would suggest a lower sensitivity of ICT compared to other serological methods and therefore provide evidence for the need to replace ICT as the screening test.

**TABLE 3:** Results of different serological methods for the detection of *Trypanosoma cruzi* antibodies in CD patients

Patient ID	ICT result	CLIA result	CLIA index	ELISA1 result	ELISA1 index	ELISA2 result	ELISA2 index	WB1 result	WB2 result
4278	neg	neg	0.02	neg	0.04	neg	0.30	neg	neg
4277	neg	pos	1.23	pos	3.05	pos	1.43	pos^a^	pos^b^
4293	pos	pos	9.37	pos	9.34	pos	3.42	pos	pos
4658	pos	pos	9.91	pos	6.75	pos	3.36	pos	pos
4287	pos	pos	11.22	pos	9.52	pos	3.80	pos	pos
4522	pos	pos	12.05	pos	8.21	pos	4.13	pos	pos
4302	pos	pos	12.79	pos	9.58	pos	3.67	pos	pos
4306	pos	pos	13.07	pos	9.31	pos	4.57	pos	pos
4666	pos	pos	13.28	pos	9.77	pos	3.80	pos	pos
4660	pos	pos	13.46	pos	9.52	pos	3.57	pos	pos

*Note:* The table shows the results of different serological methods for the detection of *T. cruzi* antibodies in CD patients (*N* = 10).

Abbreviations: ELISA1, ELISA using recombinant antigens; ELISA2, ELISA using lysate antigen; WB1, WB using TcF recombinant antigen; WB2, WB using lysate antigen.

^a^ Sample result equal to cut‐off.

^b^ This sample did not showed reactivity to lower molecular weight bands 21–22 and 15–16.

Taken together, comparison of results of different assays performed on samples for ICT‐seropositive donors and on samples from CD patients indicates no agreement between ICT and other assays (agreement = 45%–50%; Cohen *k* = 0) but demonstrates almost perfect to perfect agreement of other assays among themselves (agreement = 94%–100%; Cohen *k* = 0.88–1.00).

## 4 DISCUSSION

When individuals from CD endemic countries migrate to non‐endemic countries and act as donors (blood or other cellular products), there is a need to prevent transmission through transfusion or transplantation.^5^

In this way, it is possible to guarantee the safety of blood and its products, simultaneously maintaining the blood supply from candidate donors,^6^ as shown in the United States where selective *T. cruzi* screening is nearly equally effective as universal screening, but at a reduced cost.^7^

In this study, a low prevalence of seropositive individuals (0.50%) has been observed among blood donors identified to be at risk for *T. cruzi* infection after an appropriate interview. This frequency is much lower than the 3.9% previously reported from a different Italian hospital in the Lazio region among a small number of donors (*n* = 128)^8^—possibly due to different criteria used to identify at‐risk donors via questionnaire as this study anticipated the introduction of national legislation—but is in the range of results reported from other European countries such as Spain (1.91%), France (0.31%), Switzerland (0.08%), the United Kingdom (0.50%) and the Netherlands (nul).^1^

Comparison of results of different serological methods on seropositive donors and CD patients suggested lower sensitivity of ICT. This observation confirms results published when the present study was ongoing, which showed that the sensitivity of ICT was not optimal (82.8%) in non‐endemic countries.^9^ It is therefore important to acknowledge the fact that, in light of the non‐optimal sensitivity of the ICT assay used for screening, the observed prevalence of seropositive at‐risk donors might be underestimated. Furthermore, the fact that ICT‐positive results obtained on seropositive donors were not confirmed by other assays also raises concerns regarding this test's specificity.

An ELISA test based on lysate antigen would be the preferred choice as a screening test based on simplicity of the procedure, the multi‐sample format and on the need to use different antigens with respect to the second test (CLIA based on recombinant antigens).

## 5 CONCLUSION

The present study, although limited by the small number of samples used for comparison of serological methods, provides evidence that supports the need to reconsider the diagnostic algorithm for CD at reference laboratories in Italy. We suggest, for blood donor screening, that an ELISA assay based on *T. cruzi* lysate could be the first test used, and as second test, a CLIA assay based on *T. cruzi* recombinant antigens should be carried out. It is worth noting that all CD sera that tested positive with CLIA were confirmed positive with both WB tests. As the third, or confirmatory, test, we suggest a WB assay based on crude antigen.

More generally, there is a recommendation to discontinue the use of ICT as a screening test given its limited sensitivity and specificity. It is envisaged that a sensitive and multi‐sample format assay be used as a screening test and that this should be adopted at the national level for uniformity of results; a second test, as well as a confirmatory one, should be available at reference laboratories for the rapid and safe exclusion of CD in seropositive blood donors.

In addition to donor selection, other strategies may increase transfusion safety, such as pathogen inhibition methods.^10^, ^11^ These methods would lower the risk not only of TT CD but also of other parasitic diseases such as malaria, babesiosis and leishmaniosis whose importance in transfusion medicine is often neglected.^12^

## CONFLICT OF INTEREST

The authors have no competing interests.

## AUTHOR CONTRIBUTIONS

Valentina D. Mangano and Fabrizio Bruschi conceived the study. Marco Prato and Giovanna Moscato performed the experiments. Valentina D. Mangano performed data analyses. Valentina D. Mangano, Marco Prato and Fabrizio Bruschi wrote the manuscript. All authors revised and approved the final version of the manuscript.

## References

[1] Lidani KCF , Andrade FA , Bavia L , et al. Chagas disease: from discovery to a worldwide health problem. Front Public Health. 2019;7:166. 31312626 10.3389/fpubh.2019.00166 PMC6614205

[2] Pinazo MJ , Gascon J . The importance of the multidisciplinary approach to deal with the new epidemiological scenario of Chagas disease (global health). Acta Trop. 2015;151:16‐20. 26187358 10.1016/j.actatropica.2015.06.013

[3] WHO . WHO Consultation on International Biological Reference Preparations for Chagas Diagnostic Tests. Geneva, Switzerland: World Health Organization; 2007.

[4] Angheben A , Boix L , Buonfrate D , et al. Chagas disease and transfusion medicine: a perspective from non‐endemic countries. Blood Transfus. 2015;13(4):540‐550. 26513769 10.2450/2015.0040-15 PMC4624528

[5] Kitchen AD , Hewitt PE , Chiodini PL . The early implementation of *Trypanosoma cruzi* antibody screening of donors and donations within England: preempting a problem. Transfusion. 2012;52:1931‐1939. 22414025 10.1111/j.1537-2995.2012.03599.x

[6] Reynolds CA , Cieply L , Sell J , Brailsford SR . Who do we gain? Enhancement of blood supplies by additional testing for donors who travel. Transfus Med. 2019;29(5):325‐331. 31347219 10.1111/tme.12620

[7] Agapova M , Busch MP , Custer B . Cost‐effectiveness of screening the US blood supply for *Trypanosoma cruzi* . Transfusion. 2010;50:2220‐2232. 20492607 10.1111/j.1537-2995.2010.02686.x

[8] Gabrielli S , Girelli G , Vaia F , Santonicola M , Fakeri A , Cancrini G . Surveillance of Chagas disease among at‐risk blood donors in Italy: preliminary results from Umberto I polyclinic in Rome. Blood Transfus. 2013;11(4):558‐562. 24120609 10.2450/2013.0055-13 PMC3827401

[9] Angheben A , Staffolani S , Anselmi M , et al. Accuracy of a rapid diagnostic test (Cypress Chagas Quick Test[®]) for the diagnosis of chronic Chagas disease in a nonendemic area: a retrospective longitudinal study. Am J Trop Med Hyg. 2017;97(5):1486‐1488. 28820710 10.4269/ajtmh.17-0205 PMC5817761

[10] Yonemura S , Doane S , Keil S , Goodrich R , Pidcoke H , Cardoso M . Improving the safety of whole blood‐derived transfusion products with a riboflavin‐based pathogen reduction technology. Blood Transfus. 2017;15(4):357‐364. 28665269 10.2450/2017.0320-16 PMC5490732

[11] Galli L , Bruschi F . New strategies for the control of infectious and parasitic diseases in blood donors: the impact of pathogen inactivation methods. Eurobiotech J. 2020;4(2):53‐66.

[12] Leiby DA , O'Brien SF , Wendel S , et al. WPTTID subgroup on parasites. International survey on the impact of parasitic infections: frequency of transmission and current mitigation strategies. Vox Sang. 2019;114(1):17‐27. 30523642 10.1111/vox.12727

